# Long-Term Remission of a Spinal Atypical Teratoid Rhabdoid Tumor in Response to Intensive Multimodal Therapy

**DOI:** 10.1155/2019/3842835

**Published:** 2019-10-13

**Authors:** Fahd Refai, Haneen Al-Maghrabi, Hassan Al Trabolsi, Jaudah Al-Maghrabi

**Affiliations:** ^1^Department of Pathology, Faculty of Medicine, King Abdulaziz University, Jeddah, Saudi Arabia; ^2^Department of Pathology, King Faisal Specialist Hospital and Research Centre, Jeddah, Saudi Arabia; ^3^Oncology Department, King Faisal Specialist Hospital and Research Centre, Jeddah, Saudi Arabia

## Abstract

Atypical teratoid rhabdoid tumors (ATRTs) are rare and aggressive central nervous system tumors that infrequently arise in spinal locations in young children. Provided clinical and diagnostic suspicion is high, the histopathological diagnosis is relatively straightforward to secure by testing for the characteristic loss of the tumor suppressor protein *SMARCB1/INI1*. Here, we describe a case of thoracic spinal ATRT in a three-year-old boy that showed characteristic aggressive progression until managed with intensive multimodal therapy to achieve durable long-term remission. In doing so, we review the histopathological features, management, and current advances in molecular biology that hold promise for personalized ATRT therapy.

## 1. Introduction

Atypical teratoid rhabdoid tumors (ATRTs) are rare, aggressive, central nervous system (CNS) tumors that usually arise in the brain in young children but can arise at other sites including the spinal cord [1]. ATRTs need to be considered in the differential diagnosis of small round blue cells and poorly differentiated tumors occurring at peripheral sites, especially when biopsy material is limited, since the tumors are frequently heterogeneous and characteristic rhabdoid cells may be sparsely distributed. However, provided that diagnostic suspicion is high, the diagnosis of ATRT is aided by characteristic and almost pathognomonic molecular features. Here, we describe the rare case of a spinal ATRT occurring in a young boy to illustrate not only the histopathological and molecular features but also the aggressive but rescuable clinical course of ATRT with intensive multimodal management.

## 2. Case Presentation

**Figure 1:**
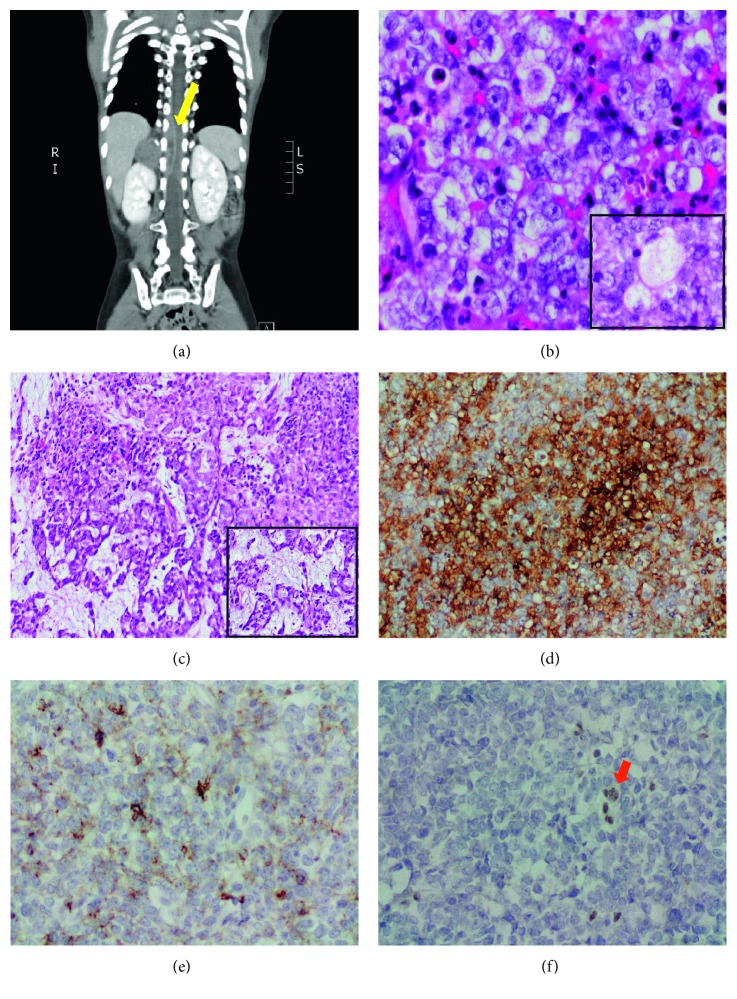
(a) Initial CT scan which shows 2.8 cm right suprarenal hypodense mass, extending to the spinal canal (T9–T12) and cause spinal cord compression (yellow arrow). (b) Histology of the tumor is composed of large pleomorphic cells with prominent nucleoli and clear nearly vacuolated cytoplasm (inset); background of scattered acute inflammatory cells are seen (Hematoxylin and eosin (H&E) stain; 40x). (c) Tumor cells containing more abundant eosinophilic cytoplasm, with focal myxoid changes (inset) (Hematoxylin and eosin (H&E) stain; 10x). (d) Tumor cells are diffusely positive for SMA (20x). (e) Focal tumor reactivity for EMA is seen (20x). (f) Complete loss of tumor cells' immunoreactivity for *SMARCB1/INI1*, with maintained internal control in lymphocytes between tumor cells (red arrow) (20x).

A three-and-a-half-year-old boy presented to King Faisal Specialist Hospital & Research Centre, Jeddah, Kingdom of Saudi Arabia, in July 2015 with a ten-day history of back pain and progressive weakness of the lower extremities and an inability to walk. A computerized tomography (CT) scan performed at that time revealed a right suprarenal hypodense mass measuring 2.8 × 3 × 4.6 cm and extending to the spinal canal (T9–T12) to cause spinal cord compression (Figure 1(a)). He underwent urgent laminectomy and decompression followed by debulking one month later, which improved his neurological symptoms. Histopathological examination at that time revealed a highly cellular tumor composed of cells with large nuclei, prominent nucleoli, and a variable amount of cytoplasm. Some of the cells had clear, nearly vacuolated cytoplasm (Figure 1(b)), and scattered cells contained more abundant, eosinophilic cytoplasm, but there were no classic rhabdoid cells with accumulation of intermediate filaments (Figure 1(c)). Immunohistochemistry for antibodies targeting vimentin, smooth muscle actin alpha (SMA) (Figure 1(d)), and p63 was positive, and there was focal positivity for Bcl-2 and focal weak positivity for CD99, CD10, CK8/18, calponin, and epithelial membrane antigen (EMA) (Figure 1(e)). Tumor cells were negative for other immune cell, neural, epithelial cell, germ cell, and smooth muscle cell markers. However, there was loss of tumor cell immunoreactivity for *SMARCB1/INI1* (Figure 1(f)), and Ki-67 was positive in >50% of tumor cells. Fluorescence *in situ* hybridization for EWS/FLI-1 (Ewing sarcoma/PNET) and SYT/SSX (poorly differentiated synovial carcinoma) were all negative. A diagnosis of a malignant poorly differentiated round cell neoplasm was made, and the specific differential diagnosis including atypical teratoid rhabdoid tumor (ATRT) and myoepithelial carcinoma was also made. The case was referred for an external specialist opinion at Boston Children's Hospital, who agreed with the histopathological assessment that both the morphology and the immunophenotype were consistent with ATRT, the polyphenotypic appearance of the tumor (especially EMA and smooth muscle actin positivity), and the definite loss of *SMARCB1/INI1* expression supporting the diagnosis.

Initial tumor regrowth after surgery was rapid, so the patient received 10 sessions of spinal radiotherapy and dexamethasone IV on a tapering dose in Saudi Arabia. He then went to the USA in September 2015, where he received 29 Gy radiotherapy plus dexamethasone and eight cycles of alternating vincristine, doxorubicin, cyclophosphamide, ifosfamide, cisplatin, and etoposide. He completed chemotherapy in April 2016. While surgical intervention was preferred for local control, this was not possible due to the tumor extent proximity to the spinal cord, so he underwent further 4500 cGy radiotherapy in 25 fractions to the paraspinal area in February and March 2016. A positron emission tomography (PET) scan performed after completion of therapy in 2016 was negative. A follow-up CT scan of the abdomen and pelvis in April 2017 revealed the development of a new soft tissue deposit at the inner aspect of the right 11^th^ rib suggestive of recurrent disease. He had a further course of radiotherapy and chemotherapy which resolved the recurrence, and regular pediatric oncology follow-up with imaging identified no new recurrences over the following eighteen months.

## 3. Discussion

Atypical teratoid rhabdoid tumor (ATRT) is a rare and aggressive CNS tumor. ATRTs most frequently arise in the posterior cranial fossa (especially at the cerebello-pontine angle) of young children and infants aged under three, with a slight male predominance [1]. However, ATRTs can also rarely arise in spinal locations, as seen here, and also the kidneys and other peripheral, intrabdominal, and retroperitoneal sites [1]. Although in close proximity to the kidney, there was no renal parenchymal involvement of the ATRT described here, so we consider this to be an example of a spinal ATRT, of which about 40 are described in the literature [1–3]. Originally called malignant rhabdoid tumors and described as aggressive variants of Wilms' tumors with rhabdomyosarcomatous features, Rorke et al. [4] fully defined the entity in 1996 before its entry into the World Health Organization (WHO) classification in 2000 [5]. Prior to this, ATRTs were commonly misdiagnosed as medulloblastomas or primitive neuroectodermal tumors (PNETs), because of not only the similar histopathological appearances but also the similar imaging and gross pathological features [5]. The histological features are often mixed. It is not unusual for ATRTs to show minimal rhabdoid features and a predominant undifferentiated blue cell appearance, as seen in this case. Approximately, 30% of tumors contain malignant mesenchymal elements and 25% glandular or squamous epithelial components, although these were not seen here. Accordingly, the immunoreactivity is similarly varied and depends on the tumor composition, but this case showed the fairly typical pattern of vimentin, EMA, and SMA positivity, a high Ki-67 proliferation index, and negativity for desmin and germ cell markers. Critically, this case exhibited loss of *SMARCB1/INI1* immunoreactivity, which corresponds to either homozygous deletions or mutations in *SMARCB1/INI1*, a tumor suppressor gene located on chromosome 22 (22q11.2) [6]. *SMARCB1/INI1* is a core subunit of the epigenetic ATP-dependent SWI/SNF chromatin remodeling complex, which is essential for survival and dysregulation of which impacts several important oncogenic pathways involved in proliferation and apoptosis including the p16-RB, WNT, sonic hedgehog, and polycomb pathways [7]. *SMARCB1/INI1* is therefore a *bone fide* tumor suppressor gene and likely driver mutation in ATRTs. *SMARCB1/INI1* is mutated in over 90% of ATRTs (with those tumors preserving *SMARCB1/INI1*, showing loss of the related SWI/SNF complex protein SMARCA4/BRG1) and is only rarely described in other CNS tumors, although is frequently lost in epithelioid sarcoma, pancreatic undifferentiated rhabdoid carcinoma, and epithelioid schwannoma [8], making it an extremely helpful ancillary test in these pediatric brain tumor cases [7]. Indeed, prior to the development of the antibody targeting *SMARCB1/INI1*, misdiagnosis was especially common if the characteristic rhabdoid cells were not present in the biopsy specimen [9]. Although usually sporadic, germline *SMARCB1/INI1* mutations have been described in as many as 35% of cases that give rise to more extensive disease in very young patients [8]. Given that *SMARCB1/INI1* is germline lethal, these germline mutations tend to arise *de novo* and hereditary cases are extremely rare [10].

The prognosis of ATRT has generally been regarded as appalling; historically, the majority of patients died within a year of diagnosis [1]. Indeed, our case highlights the rapidly progressive natural disease course in the absence of aggressive multimodal therapy, with the tumor rapidly regrowing after initial debulking. Given the rarity of the tumor and relatively few prospective trials, there has yet to be a consensus on the optimal management of ATRT. Different multimodal strategies combining surgery, radiotherapy, and chemotherapy (conventional and high dose, systemic and intrathecal, and with or without stem cell support) have been used with variable success, and one-year progression-free and overall survival rates have now improved to >50% [11–13]. Nevertheless, five-year survival rates remain only ∼30% in pooled analyses [13]. There is now good evidence that while ATRTs are chemosensitive, in order to overcome the early emergence of resistance (usually in the first 24 weeks of diagnosis), the chemotherapy regimen must be intensive and use multiple agents in a dose-dense regimen after surgery and in combination with radiotherapy. Here, we employed a modification of the Medical University of Vienna protocol, which in a retrospective analysis achieved a 100% 5-year overall survival rate and 88.9% event-free survival rate [14]. Although our patient is now approaching four years since the diagnosis, there are no reliable prognostic markers for ATRT, and he will require continuing and close follow-up. With respect to prognosis, recent molecular profiling efforts are starting to define ATRT heterogeneity, with ASCL1 arising risk factors with clinicopathological characteristics and outcomes (supratentorial, improved overall survival) [15]. Of note, ASCL1+ tumors had superior radiation-free survival in this analysis, raising the prospect of a predictive biomarker that might help personalize therapy and spare some patients unnecessary therapies and consequent side effects.

In summary, the characteristic *SMARCB1/INI1* mutations seen in ATRT have facilitated its histopathological diagnosis. However, the wider clinical and molecular heterogeneity of ATRT remains unresolved, and further efforts are required to develop prognostic and predictive biomarkers to personalize therapy for these young children. While there has been significant progress in the multimodal management of ATRT, there remains a need for coordinated, international, multicenter efforts to standardize management in the context of prospective clinical trials with associated molecular analyses.
